# Fluorescence Analysis of Local Microenvironments in Polymer Films Using Solvatochromic Dyes

**DOI:** 10.3390/s26041346

**Published:** 2026-02-20

**Authors:** Tomoharu Matsushita, Takuya Tanaka, Yuki Sawatari, Gen-ichi Konishi

**Affiliations:** Department of Chemical Science and Engineering, Institute of Science Tokyo, Tokyo 152-8552, Japansawatari.y.aa@m.titech.ac.jp (Y.S.)

**Keywords:** solvatochromic dye, intramolecular charge transfer (ICT), ratiometric fluorescence thermometry, polymer films, polymer microenvironment, fluorescence lifetime (TCSPC), local polarity, structural relaxation, microheterogeneity

## Abstract

Polymer films and polymer blend films are widely used as functional materials; however, their photophysical behavior cannot be fully explained solely by bulk properties such as relative permittivity or glass transition temperature. In this study, we investigate how local polymer microenvironments regulate fluorescence responses by employing two strongly emissive solvatochromic dyes—**FπPCM**, a D–π–A-type π-conjugation-extended fluorene dye, and **PK**, a D–π–A-type pyrene dye—as molecular probes. The photophysical properties of these dyes were systematically examined in a series of transparent polymer matrices, including polystyrene, polycarbonate, poly(methyl methacrylate), poly(vinyl chloride), triacetylcellulose, poly(butyl methacrylate), and poly(2-ethyl-2-oxazoline). Polymer films containing the dyes were prepared by solution casting from homogeneous polymer–dye solutions onto quartz substrates followed by solvent evaporation. Both dyes exhibited polymer-dependent variations in fluorescence wavelength, quantum yield, and lifetime, reflecting not only differences in polymer polarity but also local chain packing and specific dye–polymer interactions. Fluorescence lifetime analysis of PS/POz blend films revealed microscopic heterogeneity even in miscible systems, quantitatively captured using averaged lifetime parameters. Temperature-dependent fluorescence measurements further demonstrated that thermal history and structural relaxation significantly influence local polymer environments. In particular, ratiometric fluorescence analysis of PMMA/PBMA blend films enabled reproducible temperature sensing over a wide range from 30 to 120 °C, despite an overall negative temperature response. These results establish solvatochromic dyes as versatile optical probes for evaluating local polymer microenvironments and highlight their potential for polymer-state monitoring and fluorescence-based temperature-sensing applications.

## 1. Introduction

Polymer films and polymer blend films are widely used in diverse applications such as optical waveguides [1] and solar cells [2]. However, their functional performance cannot always be rationalized solely by bulk parameters such as relative permittivity (*ε*_r_), glass transition temperature (*T*_g_), and elastic modulus. In practical systems, fluorescence behavior is strongly influenced by the local environments surrounding embedded molecules, including local polarity, segmental mobility of polymer chains, microscopic heterogeneity (e.g., local composition fluctuations that may arise even in thermodynamically miscible systems), and structural relaxation associated with thermal history and annealing. Therefore, experimental strategies capable of probing polymer microenvironments under realistic operating conditions are increasingly required.

For polymer film characterization, various analytical techniques have been widely employed. Differential scanning calorimetry (DSC) has been extensively used to evaluate *T*_g_ and miscibility in polymer blends through composition-dependent *T*_g_ shifts [3,4,5]. Dynamic mechanical analysis (DMA) provides complementary information by enabling the detection of phase separation that may not be observable by DSC and allowing quantitative evaluation of viscoelastic relaxation dynamics, including storage modulus evolution and free-volume-related parameters [6,7,8]. Structural ordering and crystallinity have been investigated using X-ray diffraction (XRD) and grazing-incidence (GI) scattering techniques [9,10,11], while atomic force microscopy (AFM) has been widely applied to visualize surface morphology and nanoscale phase-separation features in polymer films [12,13]. Despite these advances, most techniques provide bulk-averaged or static structural information and do not directly reflect excited-state environments experienced by emissive probes, such as local polarity, specific intermolecular interactions, and local dynamics. This limitation becomes particularly significant in polymer blends, where multiple microenvironments can coexist on microscopic length scales, making it difficult to quantitatively rationalize emission behavior using a single *ε*_r_ or a single *T*_g_ value. Moreover, non-destructive optical methods capable of tracking dynamic structural changes induced by thermal history remain limited.

In recent years, fluorescent dyes have attracted increasing attention not only as sensitive and non-destructive probes in bioimaging [14,15,16,17,18,19,20,21,22,23], but also as molecular reporters embedded in condensed-phase materials such as polymer films and gels, where environmental responsiveness can be exploited to visualize internal material states [24,25,26,27,28,29,30,31]. In polymer films, fluorescent probes have been widely used to report local polarity and specific intermolecular interactions through solvatochromic emission shifts [32,33,34]. In addition, macromolecular orientation and packing of polymer matrices can modulate emission wavelengths and spectral shapes via anisotropic microenvironments and dye alignment effects [35]. In gels, fluorescence probes have enabled visualization of phase transitions and local suppression of molecular motion, providing optical access to dynamic microenvironments [36,37]. Furthermore, mechanoresponsive fluorescence responses, including mechanofluorochromism arising from aggregate reorganization or mechanophore activation, have been reported [38,39,40]. These studies highlight fluorescence spectroscopy as a versatile optical sensing strategy for probing condensed-phase microenvironments.

Among these probes, solvatochromic dyes are particularly attractive because they respond sensitively to environmental polarity and report excited-state stabilization—especially of intramolecular charge-transfer (ICT) states—through changes in fluorescence wavelength, quantum yield, and lifetime [41,42]. Such multidimensional optical responses make solvatochromic dyes promising candidates for quantitative sensing of polymer microenvironments. Building on our recent findings that temperature-dependent changes in solution can be effectively captured by fluorescence measurements [43], solvatochromic dyes offer a promising platform for optical evaluation of local environments and their temperature responsiveness in polymer films.

In this study, we employed two strongly emissive solvatochromic dyes previously reported by our group: **FπPCM**, a D–π–A-type π-conjugation-extended fluorene dye [44,45], and **PK** [46,47,48,49], a D–π–A-type pyrene dye. Their photophysical properties—including fluorescence wavelength, quantum yield, and fluorescence lifetime—were systematically compared in a series of transparent polymer matrices, namely polystyrene (PS), polycarbonate (PC), poly(methyl methacrylate) (PMMA), poly(vinyl chloride) (PVC), triacetylcellulose (TAC), poly(butyl methacrylate) (PBMA), and poly(oxazoline) (POz). Furthermore, using PS/POz blend films as a model system, we discuss how microscopic heterogeneity in miscible blends influences fluorescence lifetime behavior based on averaged lifetime analysis. We also investigate thermal-history effects and demonstrate ratiometric fluorescence thermometry in PMMA/PBMA blend films, extending solvatochromic dyes beyond conventional polarity probes toward optical sensing of polymer microenvironment dynamics and temperature.

## 2. Materials and Methods

### 2.1. Materials and Sample Preparation

In this study, commonly used transparent polymer matrices were employed, including polystyrene (PS, *M*_w_ = 35,000, Sigma-Aldrich (Tokyo, Japan)), polycarbonate (PC, BPA-PC, *M*_w_ = 64,000, Sigma-Aldrich (Tokyo, Japan)), poly(methyl methacrylate) (PMMA, *M*_w_ = 100,000, FUJIFILM Wako Pure Chemical Corporation (Tokyo, Japan)), poly(vinyl chloride) (PVC, *M*_w_ = 50,000–75,000, Kanto Chemical Co., Inc. (Tokyo, Japan)), triacetylcellulose (TAC, FUJIFILM Wako Pure Chemical Corporation (Tokyo, Japan)), poly(butyl methacrylate) (PBMA, *M*_w_ = 200,000, Sigma-Aldrich (Tokyo, Japan)), and poly(2-ethyl-2-oxazoline) (POz, *M*_w_ ≈ 50,000, Sigma-Aldrich (Tokyo, Japan)). The dye concentration in all polymer films was adjusted to 0.1 wt%. Each polymer and dye were completely dissolved in the corresponding solvent to obtain homogeneous solutions prior to film preparation. PS, BPA-PC, PMMA, PVC, PBMA, and POz were dissolved in tetrahydrofuran (THF), whereas TAC was dissolved in a mixed solvent of dichloromethane/methanol (9/1, *v*/*v*). The resulting solutions were cast onto quartz substrates and allowed to dry under ambient conditions to achieve slow solvent evaporation, affording uniform polymer films.

The film thickness was not precisely measured; however, all samples were prepared using identical solution concentrations and casting volumes, resulting in comparable optical densities across different polymer matrices. Because the dye concentration was fixed at 0.1 wt%, the local polymer environment surrounding each dye molecule is expected to be governed primarily by the intrinsic polymer properties rather than macroscopic film thickness variations. To minimize solvent effects on fluorescence properties, the films were dried thoroughly until no further spectral changes were observed. Therefore, the measured fluorescence parameters are considered to reflect the intrinsic polymer microenvironment rather than residual solvent contributions.

Since PS and POz are known to be mutually miscible [50], blend films of PS and POz with mass ratios of 3:1, 1:1, and 1:3 were also prepared. For the PS–POz blend samples, PS and POz were first dissolved in chloroform, followed by reprecipitation by dropwise addition into hexane cooled to 0 °C. The resulting precipitates were collected and dried under vacuum prior to film preparation, and films were subsequently prepared using the same casting procedure described above.

### 2.2. Photophysical Measurements

Fluorescence lifetimes in solution were measured using an Edinburgh Instruments FS5 spectrofluorometer equipped with a time-correlated single-photon counting (TCSPC) system and an EPL-405 picosecond pulsed diode laser (*λ*_ex_ = 402.5 ± 5 nm; pulse width ≈ 75 ps; repetition rate 2.5 kHz–20 MHz). Absolute photoluminescence quantum yields were determined using a Hamamatsu Photonics Quantaurus-QY system. Temperature-dependent fluorescence spectra of polymer films were measured using an Edinburgh Instruments FS5 spectrofluorometer equipped with an SC-50 Optical Fibre Launcher, coupled to a polarized optical microscope (POM; Olympus BX51) and a METTLER TOLEDO FP82HT hot stage controlled by a METTLER TOLEDO FP900 central processor (METTLER TOLEDO, Greifensee, Switzerland).

## 3. Results

To elucidate how local polymer microenvironments govern the photophysical properties of solvatochromic dyes, we first examine their fluorescence responses in single-component polymer films with systematically varied polarity and chain characteristics. This analysis establishes a baseline framework for interpreting how polymer matrices influence fluorescence wavelength, quantum yield, and lifetime. Building on this foundation, we extend the investigation to polymer blend systems, where microscopic heterogeneity can emerge even under thermodynamically miscible conditions, and evaluate its impact on excited-state dynamics. Finally, we investigate temperature-dependent fluorescence behavior to clarify the effects of thermal history and structural relaxation and to demonstrate the feasibility of ratiometric fluorescence thermometry in polymer blend films.

### 3.1. Environment-Dependent Fluorescence Properties in Polymer Films

**Figure 1:**
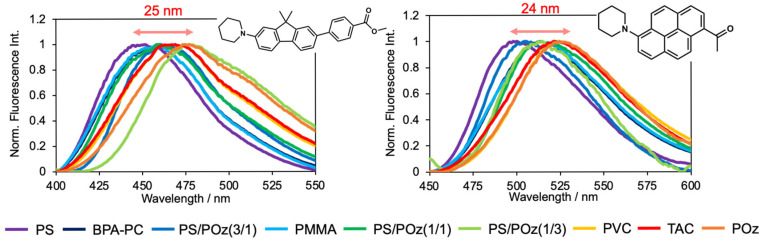
Molecular structures of the dyes and fluorescence spectra recorded in various polymer films with a dye loading of 0.1 wt%. The left panel shows the data for **FπPCM**, while the right panel corresponds to **PK**.

Table 1 summarizes the fluorescence properties of the solvatochromic dyes **FπPCM** and **PK** embedded in various polymer films, and the corresponding fluorescence spectra are shown in Figure 1. Both dyes exhibit polymer-dependent variations in the maximum fluorescence wavelength (*λ*_fl_), fluorescence quantum yield (*Φ*_fl_), and fluorescence lifetime (*τ*_fl_). For comparison, the intrinsic photophysical properties of **FπPCM** and **PK** are also summarized in Table S1. In this study, the polarity of the polymer matrices is discussed in terms of relative permittivity (*ε*_r_) [51].

For **FπPCM**, the fluorescence maximum gradually red-shifts from 452 nm in the relatively low-polarity polymer PS (*ε*_r_ = 2.4–2.7) to 477 nm in polymer matrices containing POz, which exhibits a higher relative permittivity (*ε*_r_ = 4–6). In contrast, **PK** emits at 500 nm in PS, while a further red-shift to 524 nm is observed in PVC-, TAC-, and POz-based matrices. Notably, both dyes exhibit comparable spectral shifts of approximately 25 nm across the polymer series. The fluorescence maxima of **FπPCM** in POz coincide with those observed in low-polarity solvents such as n-hexane and toluene, whereas the emission maxima of **PK** in POz are comparable to those in medium- to high-polarity solvents such as THF and dichloromethane. These results indicate that the observed fluorescence wavelengths (477 nm for **FπPCM** and 524 nm for **PK**) do not primarily reflect intrinsic differences in the intramolecular charge-transfer (ICT) character of the dyes themselves; instead, they are governed by how effectively the polymer matrix stabilizes the ICT excited state within the local dye environment. This interpretation is further supported by the *Φ*_fl_ data. In relatively rigid polymer films such as PS, PMMA, PVC, and TAC, both dyes maintain high *Φ*_fl_ values (0.8–1.0). In contrast, a pronounced decrease in *Φ*_fl_ is observed in POz and POz-rich PS/POz blend films, particularly for **PK**, where *Φ*_fl_ decreases to 0.18–0.37. This behavior suggests that **PK** is more strongly affected by interactions with polymer chains than **FπPCM**, leading to enhanced nonradiative deactivation.

Time-resolved fluorescence decay profiles and the corresponding exponential fitting results are shown in Figures S1 and S2. As evident from the *τ*_fl_ values listed in Table 1, **FπPCM** exhibits relatively short fluorescence lifetimes (*τ*_fl_ ≈ 1–2 ns) in most polymer films, whereas **PK** shows significantly longer lifetimes (*τ*_fl_ ≈ 4–5 ns). The latter lifetimes closely resemble those observed for **PK** in polar solvents such as THF and dichloromethane, suggesting that **PK** is relatively homogeneously dispersed and well solvated within the polymer matrices. In contrast, except in **PS**, **FπPCM** exhibits biexponential fluorescence decay behavior in most polymer films. This observation indicates that, even when the decay profiles appear nearly monoexponential, **FπPCM** experiences microscopic heterogeneity within the polymer matrices, likely arising from local variations in polymer–dye interactions, incomplete mixing on a microscopic scale, and possible partial intermolecular interactions among dye molecules within the polymer environment (Figure S5) [52,53]. Despite this heterogeneity, both dyes consistently report changes in local polymer polarity through their fluorescence responses.

It should be noted that the observed fluorescence behavior cannot be fully rationalized solely by the bulk *ε*_r_ values of the polymer matrices. Instead, the experimental results suggest that three factors—local polarity, chain mobility, and specific polymer–dye interactions—cooperatively determine excited-state stabilization and decay dynamics. For example, in relatively rigid glassy polymers such as PS and PMMA, high fluorescence quantum yields are maintained despite differences in *ε*_r_, indicating that restricted segmental motion effectively suppresses nonradiative decay pathways. In contrast, POz exhibits both higher polarity and increased chain mobility arising from its flexible oxazoline backbone and polar amide groups. This combination likely facilitates stronger local solvation of the ICT state while simultaneously allowing conformational relaxation, resulting in reduced *Φ*_fl_ and enhanced nonradiative decay, particularly for **PK**. Furthermore, the pronounced sensitivity of **PK** compared to **FπPCM** suggests stronger specific interactions between **PK** and polar polymer segments, consistent with the larger decrease in *Φ*_fl_ and changes in lifetime observed in POz-rich environments. Overall, these results demonstrate that fluorescence variations are governed by the interplay between local polarity and polymer dynamics rather than by *ε*_r_ alone.

### 3.2. Analysis of Fluorescence Lifetime Modulation in Polymer Blends

**Figure 2:**
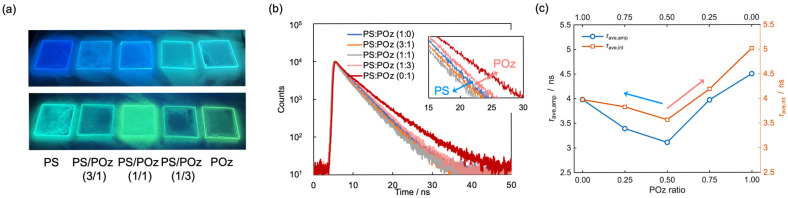
Fluorescence characteristics of PS/POz polymer blend films with varying mass fractions. (**a**) Fluorescence photographs of PS/POz blend films incorporating fluorescent dyes. Images in the upper and lower rows correspond to **FπPCM** and **PK**, respectively. The values in parentheses indicate the mass ratios of PS to POz. Excitation was performed using a black light (*λ*_ex_ = 365 nm). (**b**) Time-resolved fluorescence decay profiles of **PK** embedded in PS/POz polymer blend films at different mass fractions, recorded with an excitation wavelength of 402.5 nm. An enlarged view of the decay profiles in the 15–30 ns time window is shown. (**c**) Amplitude-averaged (*τ*_Av,amp_) and intensity-averaged (*τ*_Av,int_) fluorescence lifetimes plotted as a function of the PS/POz mass fraction, determined by exponential fitting of the decay curves shown in panel (**b**).

Next, we examine the photophysical behavior of PS/POz polymer blend films. As summarized in Table 1, the *Φ*_fl_ of **PK**, which interacts more strongly with polymer matrices than **FπPCM**, depends sensitively on the PS/POz blending ratio. Representative fluorescence photographs of the blend films at different mass fractions are shown in Figure 2a. To clarify the underlying excited-state processes, fluorescence lifetime analysis was performed.

Figure 2b shows the time-resolved fluorescence decay profiles of **PK** embedded in PS/POz blend films with varying mass fractions. The fluorescence lifetime profile increases with increasing POz content, whereas shorter lifetimes are observed as the PS fraction becomes dominant. In all PS/POz blend films, the decay curves are best described by a biexponential function (Equation (1)). Although PS and POz are thermodynamically highly miscible [54,55], these results indicate the presence of microscopic heterogeneity within the blend films, giving rise to multiple emissive microenvironments. Consequently, direct assignment of each lifetime component to a single emissive environment is not straightforward.

To address this issue, we employed the amplitude-weighted average lifetime, *τ*_Av,amp_ (Equation (2)), and the intensity-weighted average lifetime, *τ*_Av,int_ (Equation (3)), for further discussion. In heterogeneous polymer matrices, average lifetimes provide a more robust and physically meaningful descriptor of overall excited-state deactivation dynamics than individual lifetime components. The fluorescence decay profiles were fitted according to the following equations:

(1) $$R\left(t\right)=A_{1}e^{\frac{-t}{\tau_{1}}}+A_{2}e^{\frac{-t}{\tau_{2}}}$$

(2) $$\tau_{Av,amp}=\frac{\sum_{i}A_{i}\tau_{i}}{\sum_{i}A_{i}}$$

(3) $$\tau_{Av,int}=\frac{\int tR\left(t\right)dt}{\int R\left(t\right)dt}=\frac{\sum_{i}A_{i}\tau_{i}^{2}}{\sum_{i}A_{i}\tau_{i}}$$

where *A*_i_ and *τ*_i_ are the amplitude and lifetime of component *i*, respectively, *t* is the time after excitation, and *R*(*t*) represents the fluorescence intensity at time *t*. Using the fitted values of *τ*_1_ and *τ*_2_, both *τ*_Av,amp_ and *τ*_Av,int_ were calculated and plotted as a function of the PS/POz mass fraction, as shown in Figure 2c. The error propagation of the *τ*_Av,amp_ and *τ*_Av,int_ is summarized in Table S2. The standard deviations of the lifetime values were within ±0.04 ns, indicating high reliability of the fitting procedure.

As shown in Figure 2c, the dependence of both *τ*_Av,amp_ and *τ*_Av,int_ on the PS/POz composition is distinctly non-monotonic, exhibiting a clear minimum near the equimolar composition (POz = 0.50) followed by a progressive increase in the POz-rich region (POz ≥ 0.50). The initial lifetime shortening toward the 1:1 composition suggests that **PK** experiences the highest degree of microscopic heterogeneity and interfacial disorder in the mixed polymer environment, where PS-rich and POz-rich domains coexist and interfacial density is maximized. Such interfacial environments are expected to enhance nonradiative decay pathways through local packing frustration, polarity fluctuations, and dynamic disorder. Beyond this composition, both *τ*_Av,amp_ and *τ*_Av,int_ increase with increasing POz content, indicating progressive stabilization of the excited state of PK in POz-rich environments. Notably, *τ*_Av,int_ exhibits a more pronounced increase than *τ*_Av,amp_, reflecting the disproportionate contribution of longer-lived emissive populations to the time-integrated emission intensity and suggesting redistribution toward POz-dominated microenvironments.

Importantly, this lifetime elongation is accompanied by a pronounced decrease in *Φ*_fl_, as summarized in Table 1. With increasing POz content, *Φ*_fl_ of **PK** decreases systematically from 0.57 in pure PS to 0.18 in pure POz, despite the simultaneous increase in the average *τ*_fl_. This apparent decoupling between *τ*_fl_ and *Φ*_fl_ indicates that the lifetime modulation in POz-rich environments does not originate from a simple suppression of nonradiative decay; rather, it suggests a reduction in the radiative decay rate constant (*k*_r_) and/or additional nonradiative pathways induced by specific dye–polymer interactions. Such behavior is consistent with a scenario in which POz provides a more polar and hydrogen-bonding-rich local environment that stabilizes a more pronounced ICT-like excited state of **PK**. While this stabilization prolongs the excited-state lifetime, it simultaneously decreases the radiative transition probability, resulting in a lower *Φ*_fl_. Therefore, the PS/POz blending ratio acts as an effective external parameter for continuously tuning the balance between radiative and nonradiative decay processes of **PK** without altering its molecular structure. Overall, the non-monotonic lifetime behavior highlights the critical role of microenvironment heterogeneity—particularly interfacial effects—in governing excited-state dynamics and underscores the importance of evaluating both *τ*_fl_ and *Φ*_fl_ in polymer sensing systems.

### 3.3. Temperature-Dependent Photophysical Properties of Polymer Films

**Figure 3:**
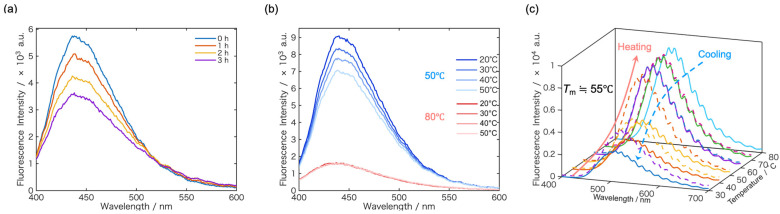
(**a**) Time evolution of the fluorescence spectra of **FπPCM** in a PBMA film during annealing at 80 °C. (**b**) Fluorescence spectra measured at various temperatures after annealing for 3 h at different annealing temperatures (50 °C and 80 °C). At each temperature, spectra were recorded after a stabilization period of 10 min. (**c**) Temperature-dependent fluorescence spectra of **FπPCM** in a PEG matrix during heating and cooling. The melting temperature of PEG is approximately 55 °C.

In this section, the temperature-dependent fluorescence behavior of polymer films was investigated using the fluorescent dye **FπPCM** as a molecular probe. As a model system, we first examined the temperature-dependent fluorescence behavior of poly(butyl methacrylate) (PBMA), whose glass transition temperature (*T*_g_) has been reported to be close to room temperature (approximately 0–30 °C) [54,55,56]. The fluorescence behavior of **FπPCM** in PBMA films was studied under thermal annealing conditions at 50 °C and 80 °C, both of which are well above the bulk *T*_g_ of PBMA (Figure 3a,b).

Time-dependent fluorescence measurements during annealing revealed a gradual decrease in fluorescence intensity with increasing annealing time (Figure 3a). This behavior suggests that **FπPCM** molecules, initially distributed in a microscopically heterogeneous manner within the as-cast PBMA film, undergo progressive reorganization upon thermal annealing. Specifically, enhanced segmental mobility of PBMA chains above *T*_g_ likely facilitates stronger and more homogeneous dye–polymer interactions, leading to modifications of the local environment surrounding **FπPCM** and enhanced nonradiative deactivation.

To further elucidate the influence of thermal history, temperature-dependent fluorescence spectra were recorded after annealing the films for 3 h at either 50 °C or 80 °C (Figure 3b). For the sample annealed at 50 °C, the fluorescence intensity decreased monotonically with increasing measurement temperature, indicating that the polymer matrix remained dynamically responsive and that thermally activated structural relaxation continued during measurement. In contrast, for the sample annealed at 80 °C, the fluorescence intensity exhibited little to no temperature dependence over the same temperature range. This marked difference suggests that annealing at 80 °C induces a more equilibrated polymer structure, in which segmental mobility of PBMA chains and the local environment surrounding **FπPCM** have already reached a thermodynamically stable state. Consequently, further temperature increases during fluorescence measurements do not significantly alter dye–polymer interactions or excited-state deactivation dynamics. These results demonstrate that fluorescence analysis provides a sensitive optical readout of thermal-history–dependent structural relaxation in polymer films, confirming that **FπPCM** serves as an effective probe for detecting subtle changes in polymer chain dynamics above *T*_g_.

### 3.4. Ratiometric Analysis of Temperature-Dependent Fluorescence in PMMA/PBMA Blend Film

**Figure 4:**
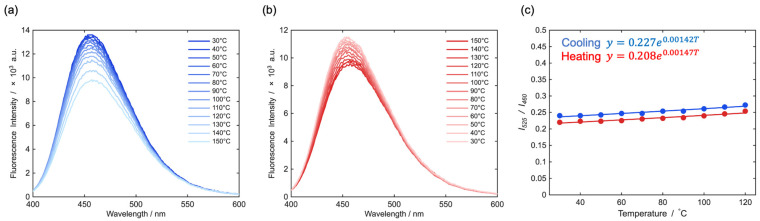
Ratiometric thermometric performance of **FπPCM** embedded in a PMMA/PBMA (1:1, *w*/*w*) film. (**a**) Temperature-dependent fluorescence spectra recorded during the heating process. (**b**) Temperature-dependent fluorescence spectra recorded during the cooling process. (**c**) Temperature dependence of the fluorescence intensity ratio at 525 nm and 460 nm (*I*_525_/*I*_460_), fitted with an exponential function. The R^2^ values are 0.950 and 0.933 for the cooling and heating processes, respectively.

In contrast to the annealing-dependent study described in Section 3.3, the temperature-dependent fluorescence behavior of PMMA/PBMA (1:1, *w*/*w*) blend films was investigated immediately after film preparation, without additional thermal annealing, to evaluate ratiometric fluorescence thermometry under nonequilibrium polymer conditions. We have previously reported that 1-(4-(9,9-dimethyl-7-(piperidin-1-yl)-9*H*-fluoren-2-yl)phenyl)-2,2,2-trifluoroethan-1-one (**FπF**), a structurally related dye bearing a trifluoroacetyl group instead of an ester substituent, exhibits a positive temperature-dependent fluorescence response in solution [43]. In the present study, the ester-substituted solvatochromic dye **FπPCM** was employed as the fluorescent probe (Figure 4).

To minimize the influence of thermal history, the PMMA/PBMA film was first heated to 150 °C and subsequently cooled to 30 °C, and the data acquired during the second heating–cooling cycle were used for analysis. Temperature-dependent fluorescence spectra were recorded at 10 °C intervals during both the heating process (Figure 4a) and the cooling process (Figure 4b). A direct comparison of the spectra indicates that the fluorescence response during cooling is less pronounced than that observed during heating; therefore, quantitative ratiometric analysis was adopted to obtain a reproducible optical readout.

Specifically, the fluorescence intensity at 460 nm (*I*_460_), corresponding to the emission maximum, and that at 525 nm (*I*_525_), where the fluorescence intensity remains nearly temperature-independent, were extracted from the spectra. The fluorescence intensity ratio, Δ(*T*) = *I*_525_/*I*_460_, was then plotted as a function of temperature. Exponential fitting of Δ(*T*) for both the heating and cooling processes yielded nearly identical fitting curves, indicating that the ratiometric response is highly reproducible and largely independent of the thermal scanning direction. Based on the obtained exponential fitting functions, the absolute thermal sensitivity (*S*_A_) and relative thermal sensitivity (*S*_R_) were calculated according to the following equations (Table 2):

(4) $$S_{A}\left(T\right)=\frac{\partial ∆\left(T\right)}{\partial T}\times 100\;\left[\%\;K^{-1}\right]$$

(5) $$S_{R}=\frac{1}{∆}\left|\frac{\partial ∆(T)}{\partial T}\right|\times 100\;\left[\%\;K^{-1}\right]$$

(6) $$\sigma \;\left(T\right)=\delta T=\frac{100}{S_{R}T}\frac{\delta ∆\left(T\right)}{∆\left(T\right)}$$

Here, Δ(*T*) represents the fluorescence intensity ratio, and σ(*T*) denotes the temperature resolution, defined as the minimum detectable temperature change determined by the relative uncertainty of the fluorescence intensity ratio, *δ*Δ(*T*)/Δ(*T*).

As summarized in Table 2, Δ(T) = *I*_525_/*I*_460_ increases monotonically with increasing temperature for both the heating and cooling processes. Because the fluorescence intensity at the emission maximum (460 nm) decreases with increasing temperature, this behavior corresponds to an overall negative temperature response. This trend contrasts with the previously reported behavior of **FπF** in solution, which exhibits a positive temperature-dependent fluorescence response. In the present study, temperature-dependent fluorescence spectra of **FπF** embedded in PMMA/PBMA (1:1, *w*/*w*) films were also recorded (Figure S4). Notably, unlike its solution behavior, **FπF** likewise exhibits a negative temperature dependence in the polymer film. This discrepancy can be attributed to restricted molecular mobility in the polymer matrix. In PMMA/PBMA films, both **FπF** and **FπPCM** experience substantially reduced conformational freedom compared to solution, suppressing the formation of highly emissive temperature-stabilized excited states. Consequently, thermally activated nonradiative decay pathways, such as internal conversion, become dominant with increasing temperature, leading to decreased fluorescence intensity. Thus, the temperature-dependent fluorescence behavior in polymer films follows the general trend observed for conventional fluorescent probes in condensed phases.

Despite the negative temperature response, Δ(*T*) could be reliably fitted by a single exponential function over a wide temperature range from 30 to 120 °C for both heating and cooling cycles. The close agreement between the fitted Δ(*T*) values and the experimental ratios, together with nearly identical fitting parameters obtained for the two thermal processes, demonstrates the robustness of the ratiometric analysis and confirms that thermal hysteresis is negligible under the present conditions. These results validate the exponential model as a practical descriptor for ratiometric fluorescence thermometry in polymer matrices.

From the exponential fits, *S*_A_ and *S*_R_ were determined. As shown in Table 2, *S*_R_ remains nearly constant at approximately 0.14% K^−1^ over the entire temperature range for both heating and cooling processes. Although this sensitivity is modest compared with systems reporting *S*_R_ values of approximately 0.18% K^−1^, numerous fluorescent thermometers in condensed-phase systems operate with comparable or lower relative sensitivities on the order of ~0.1–0.15% K^−1^ [57]. Therefore, the SR value of ~0.14% K^−1^ obtained in this study lies within a practically relevant range for temperature-sensing applications, particularly when combined with the wide operating temperature window, high reproducibility, and minimal thermal hysteresis observed here. Importantly, the nearly temperature-independent *S*_R_ represents a significant practical advantage, enabling straightforward calibration and reliable temperature readout without temperature-specific correction factors.

Notably, quantitative evaluations of *S*_R_ for organic fluorescent dyes embedded in polymer films remain relatively limited in the literature. One representative example is the rhodamine B-based polymer temperature paint reported by Yano et al., which exhibited a relative sensitivity of approximately 0.37% K^−1^ [58]. Although the *S*_R_ value obtained in the present study (~0.14% K^−1^) is smaller than that reported for the rhodamine system, the excellent fitting precision, high reproducibility, and temperature-independent stability of *S*_R_ demonstrated here indicate that the sensitivity is sufficient for reliable temperature sensing in polymer films.

In addition, the thermal resolution σ(*T*), derived from *S*_R_ and the relative uncertainty of Δ(*T*), remains nearly constant (≈6.8–7.0 °C) over the entire investigated temperature range. This uniform resolution, together with the broad operational window from 30 to 120 °C, highlights the applicability of FπPCM–PMMA/PBMA blend films as practical ratiometric fluorescent thermometers. Taken together, these results demonstrate that solvatochromic fluorescent dyes embedded in polymer films can function as robust and reproducible temperature sensors, even when the absolute sensitivity is moderate, owing to their wide dynamic range, reproducible thermal response, and minimal hysteresis.

## 4. Conclusions

In this study, we systematically investigated the photophysical behavior of strongly emissive solvatochromic dyes embedded in polymer films and polymer blend systems, focusing on how local polymer microenvironments regulate fluorescence beyond bulk-averaged material descriptors. By employing two D–π–A-type solvatochromic dyes, **FπPCM** and PK, we demonstrated that the fluorescence wavelength (*λ*_fl_), quantum yield (*Φ*_fl_), and lifetime (*τ*_fl_) are strongly modulated by the surrounding polymer matrix. These modulations reflect not only differences in relative permittivity (*ε*_r_), but also local chain packing, segmental mobility, and specific dye–polymer interactions. Accordingly, our results clearly show that bulk descriptors such as *ε*_r_ and *T*_g_ alone are insufficient to fully describe fluorescence responses in polymer films.

Fluorescence lifetime analysis of PS/POz blend films revealed that even thermodynamically miscible polymer systems exhibit microscopic heterogeneity, giving rise to multiple emissive microenvironments. The use of amplitude- and intensity-averaged lifetimes provided a robust and physically meaningful framework for capturing overall excited-state deactivation dynamics in such heterogeneous matrices. Notably, in the case of **PK**, increasing POz content led to simultaneous lifetime elongation and fluorescence quenching, indicating decoupled radiative and nonradiative processes induced by highly polar, hydrogen-bonding-rich polymer environments.

Furthermore, temperature-dependent fluorescence studies clarified the critical roles of thermal history and structural relaxation in determining polymer microenvironments. In PBMA films, annealing above *T*_g_ induced irreversible changes in fluorescence behavior, reflecting equilibration of polymer chain dynamics. In contrast, ratiometric fluorescence analysis of PMMA/PBMA blend films enabled a reproducible description of temperature dependence over a wide range (30–120 °C), despite an overall negative temperature response. The nearly constant relative sensitivity and minimal thermal hysteresis demonstrate the robustness and practical applicability of this strategy for fluorescence-based temperature sensing.

Overall, this work demonstrates that solvatochromic dyes can serve as sensitive molecular probes for visualizing polymer microenvironments and their dynamic evolution. Beyond temperature sensing, the present findings provide quantitative optical readouts correlated with polymer microheterogeneity and relaxation dynamics, highlighting the potential of fluorescence-based approaches for polymer-state monitoring and functional material sensing applications.

## Figures and Tables

**Table 1:** Maximum fluorescence wavelength (*λ*_fl_), absolute fluorescence quantum yield (*Φ*_fl_), and fluorescence lifetime (*τ*_fl_) of **FπPCM** and **PK** in various polymer films. Fluorescence lifetime measurements were performed with an excitation wavelength of 402.5 nm and an instrument response function of 78 ps.

Entry	*ε*_r_ ^1^	FπPCM			PK		
		*λ*_fl_/nm	*Φ*_fl_	*τ*_fl_/ns (%) ^2^	*λ*_fl_/nm	*Φ*_fl_	*τ*_fl_/ns (%) ^2^
PS	2.4–2.7	452	0.87	2.01	500	0.57	3.98
BPA-PC	3.2	459	0.96	1.03 (21) 1.92 (79)	515	0.85	4.83
PS/POz (3/1)	-	460	0.68	-	505	0.56	1.27 (25) 4.09 (75)
PMMA	3.6	459	0.94	1.21 (36) 2.17 (64)	515	0.89	4.99
PS/POz (1/1)	-	459	0.91	-	515	0.37	1.60 (38) 4.05 (62)
PS/POz (1/3)	-	477	0.42	-	515	0.30	1.56 (13) 4.34 (87)
PVC	3.39–3.5	469	0.99	1.27 (30) 2.18 (70)	524	0.95	5.35
TAC	3.0–4.5	469	0.84	1.84 (93) 4.11 (7.0)	522	0.94	5.14
POz	-	477	0.92	1.31 (41) 2.45 (59)	524	0.18	2.11 (29) 5.47 (71)

^1^ Relative permittivity (*ε*_r_) values used in this study were taken from Ref. [51]. ^2^ Amplitudes (fractional contributions) for each decay component derived from biexponential fitting according to Equation (1).

**Table 2:** Temperature dependence of the fluorescence intensity ratio (*I*_525_/*I*_460_), thermometric parameter Δ(*T*), absolute thermal sensitivity (*S*_A_), relative thermal sensitivity (*S*_R_), and thermal resolution σ(*T*) for **FπPCM** embedded in PMMA.

	Cooling Process				
Temp./°C	Ratio	Δ*T*	*S*_a_ (*T*)/%°C^−1^	*S*_r_ (*T*)/%°C^−1^	*σ* (*T*)
30	0.240	0.237	0.034	0.142	7.03
40	0.240	0.240	0.034	0.142	7.03
50	0.242	0.243	0.035	0.142	7.03
60	0.247	0.247	0.035	0.142	7.03
70	0.247	0.250	0.036	0.142	7.03
80	0.254	0.254	0.036	0.142	7.03
90	0.254	0.258	0.037	0.142	7.03
100	0.261	0.261	0.037	0.142	7.03
110	0.266	0.265	0.038	0.142	7.03
120	0.273	0.269	0.038	0.142	7.03
	**Heating Process**				
	**Ratio**	**Δ*T***	***S*_a_** **(*T*)/%°C^−1^**	***S*_r_** **(*T*)/%°C^−1^**	***σ*** **(*T*)**
30	0.220	0.218	0.032	0.147	6.81
40	0.223	0.221	0.032	0.147	6.81
50	0.223	0.224	0.033	0.147	6.81
60	0.225	0.227	0.033	0.147	6.81
70	0.230	0.231	0.034	0.147	6.81
80	0.232	0.234	0.034	0.147	6.81
90	0.234	0.238	0.035	0.147	6.81
100	0.240	0.241	0.035	0.147	6.81
110	0.245	0.245	0.036	0.147	6.81
120	0.254	0.248	0.036	0.147	6.81

## Data Availability

The original contributions presented in the study are included in the article/Supplementary Materials. Further inquiries can be directed to the corresponding author.

## Supplementary Materials

The following supporting information can be downloaded at: https://www.mdpi.com/article/10.3390/s26041346/s1. Figures S1–S5: Tables S1–S3. Table S1. Photophysical properties of **FπPCM** and **PK** in various organic solvents. Figure S1. Fluorescence decay curves and fitting results of **FπPCM** in polymer films. Measurements were carried out with an excitation wavelength of 402.5 nm. The same fitting range was applied to all polymer films, and the decay profiles were analyzed using a tail-fitting approach. Figure S2. Fluorescence decay curves and fitting results of **PK** in polymer films. Measurements were carried out with an excitation wavelength of 402.5 nm. The same fitting range was applied to all polymer films, and the decay profiles were analyzed using a tail-fitting approach. Table S2. Fluorescence lifetime parameters of **FπPCM** and **PK** in various polymer films obtained from the analyses of Figures S1 and S2. The fluorescence decay curves were fitted using single- or biexponential functions, yielding lifetimes (τ_1_, τ_2_), their associated amplitudes (A_1_, A_2_), amplitude-averaged lifetimes (τ_Av,amp_), and intensity-averaged lifetimes (τ_Av,int_). Errors represent fitting uncertainties, and χ^2^ values indicate the goodness of fit. Figure S3. Fluorescence decay curves and fitting results of PS/POz in polymer films. Measurements were carried out with an excitation wavelength of 402.5 nm. The same fitting range was applied to all polymer films, and the decay profiles were analyzed using a tail-fitting approach. Table S3. Fluorescence lifetime parameters of **FπPCM** in PS/POz polymer blend films obtained from the analyses of Figures S3. The fluorescence decay curves were fitted using single- or biexponential functions, yielding lifetimes (τ_1_, τ_2_), their associated amplitudes (A_1_, A_2_), amplitude-averaged lifetimes (τ_Av,amp_), and intensity-averaged lifetimes (τ_Av,int_). χ^2^ values indicate the goodness of fit. Figure S4. Temperature-dependent fluorescence spectra of **FπF** embedded in a PBMA film, recorded during heating. Figure S5. Fluorescence lifetime decay profiles of **FπPCM** embedded in **PMMA** films at different dye concentrations (0.1, 1.0, and 10 wt%).
