# Supplementary material for: Fluorescence Analysis of Local Microenvironments in Polymer Films Using Solvatochromic Dyes
Source: Sensors (Basel). 2026 Feb 20;26(4):1346. doi: 10.3390/s26041346 (PMC12944192; doi:10.3390/s26041346)
Supplement: Supplementary file 1 [file sensors-26-01346-s001.zip › sensors-4152052-supplementary.pdf]

Supporting Information for

Fluorescence Analysis of Local Microenvironments in Polymer Films Using Solvatochromic Dyes

Table S1.Photophysical properties of **FπPCM** and **PK** in various organic solvents.

Entry	FπPCM		PK	
	λ_{fl} / nm	Φ_{fl}	λ_{fl} / nm	Φ_{fl}
cyclohexane	434	0.94	480	0.74
diethyl ether	471	0.95	504	0.80
1,4-dioxane	473	0.99	514	0.89
ethyl acetate	508	0.89	524	0.72
THF	515	0.91	528	0.79
dichloromethane	547	0.91	538	0.83
acetonitrile	570	0.84	549	0.76
DMF	594	0.94	557	0.78

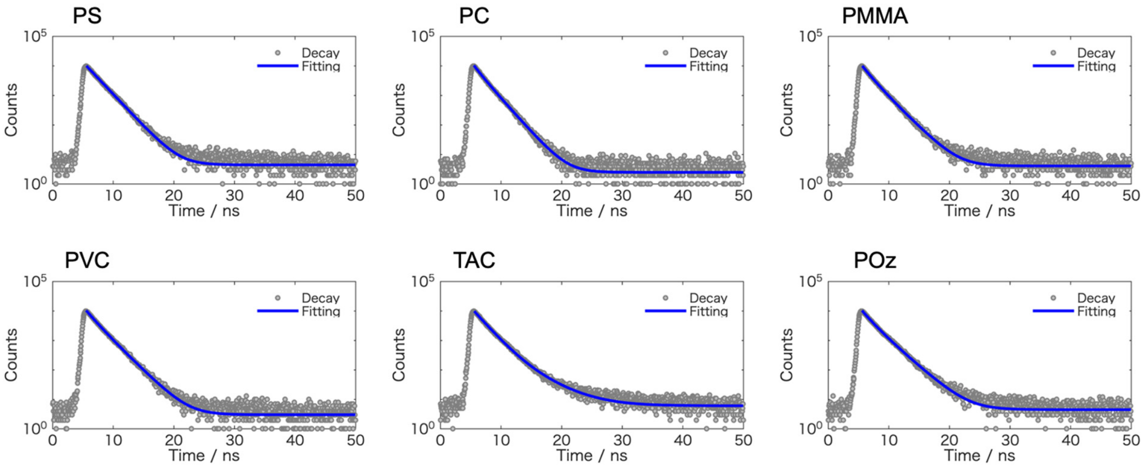

Figure S1. Fluorescence decay curves and fitting results of **FπPCM** in polymer films. Measurements were carried out with an excitation wavelength of 402.5 nm. The same fitting range was applied to all polymer films, and the decay profiles were analyzed using a tail-fitting approach.

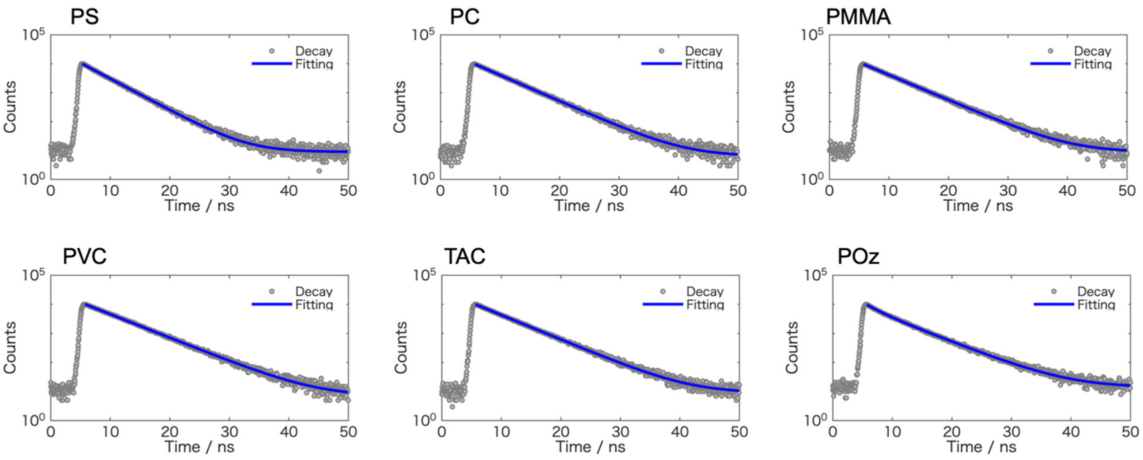

Figure S2. Fluorescence decay curves and fitting results of **PK** in polymer films. Measurements were carried out with an excitation wavelength of 402.5 nm. The same fitting range was applied to all polymer films, and the decay profiles were analyzed using a tail-fitting approach.

Table S2. Fluorescence lifetime parameters of **FπPCM** and **PK** in various polymer films obtained from the analyses of **Figures S1** and **S2**. The fluorescence decay curves were fitted using single- or biexponential functions, yielding lifetimes (τ_1, τ_2), their associated amplitudes (A_1, A_2), amplitude-averaged lifetimes ($\tau_{Av,amp}$), and intensity-averaged lifetimes ($\tau_{Av,int}$). Errors represent fitting uncertainties, and χ^2 values indicate the goodness of fit.

		τ_1 / ns	τ_1 Err / ns	A_1	τ_2 / ns	τ_2 Err / ns	A_2	τ_{AvAmp} / ns	τ_{AvAmp} Err / ns	τ_{AvInt} / ns	τ_{AvInt} Err / ns	χ^2
FπPCM	PS	2.01	0.00	10241				2.01	0.00	2.01	0.00	1.723
	PC	1.03	0.08	2289	1.92	0.02	8398	1.73	0.03	1.80	0.02	1.306
	PMMA	1.21	0.06	3740	2.17	0.03	6586	1.82	0.04	1.94	0.03	1.261
	PVC	1.27	0.08	3179	2.18	0.03	7567	1.91	0.04	2.00	0.03	1.283
	TAC	1.84	0.01	9356	4.11	0.13	735	2.00	0.02	2.18	0.04	1.459
	Poz	1.31	0.05	4310	2.45	0.03	6315	1.99	0.03	2.15	0.03	1.485
PK	PS	3.98	0.00	10097				3.98	0.00	3.98	0.00	1.317
	PC	4.83	0.01	9871				4.83	0.01	4.83	0.01	1.164
	PMMA	4.99	0.01	9792				4.99	0.01	4.99	0.01	1.162
	PVC	5.35	0.01	10167				5.35	0.01	5.35	0.01	1.161
	TAC	5.14	0.01	10022				5.14	0.01	5.14	0.01	1.165
	POz	2.13	0.07	2861	5.48	0.03	6975	4.51	0.04	5.02	0.03	1.339

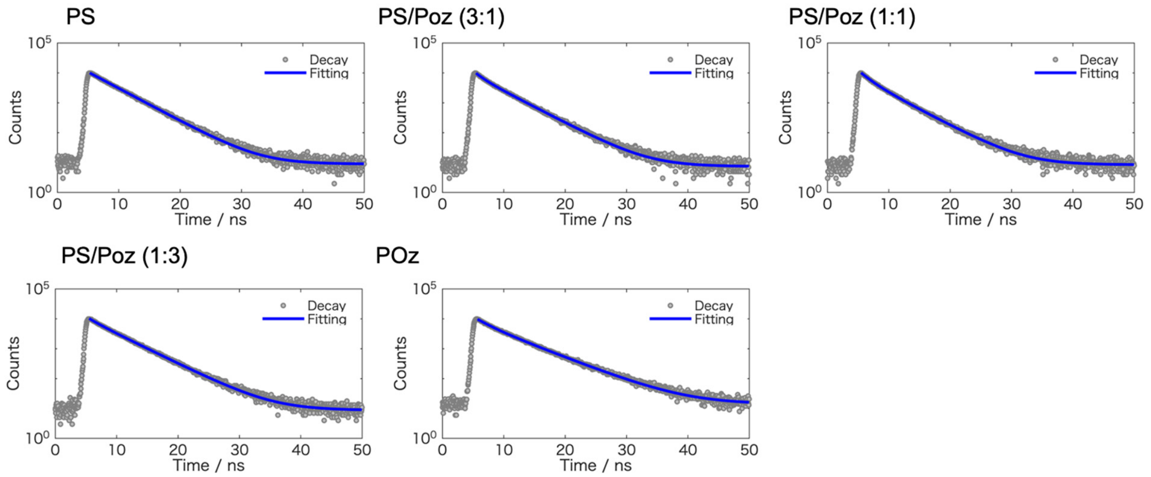

Figure S3. Fluorescence decay curves and fitting results of **PS/POz** in polymer films. Measurements were carried out with an excitation wavelength of 402.5 nm. The same fitting range was applied to all polymer films, and the decay profiles were analyzed using a tail-fitting approach.

Table S3. Fluorescence lifetime parameters of **FπPCM** in PS/POz polymer blend films obtained from the analyses of **Figures S3**. The fluorescence decay curves were fitted using single- or biexponential functions, yielding lifetimes (τ_1, τ_2), their associated amplitudes (A_1, A_2), amplitude-averaged lifetimes ($\tau_{Av,amp}$), and intensity-averaged lifetimes ($\tau_{Av,int}$). χ^2 values indicate the goodness of fit.

PS/Poz	τ_1 / ns	A_1 / %	τ_2 / ns	A_2 / %	τ_{AvAmp} / ns	τ_{AvInt} / ns	χ^2
1:0	3.98	100			3.98	3.98	1.317
3:1	1.27	25	4.09	75	3.39	3.83	1.101
1:1	1.60	38	4.05	62	3.11	3.57	1.226
1:3	1.56	13	4.34	87	3.97	4.19	1.037
1:0	2.11	29	5.47	71	4.5	5.02	1.339

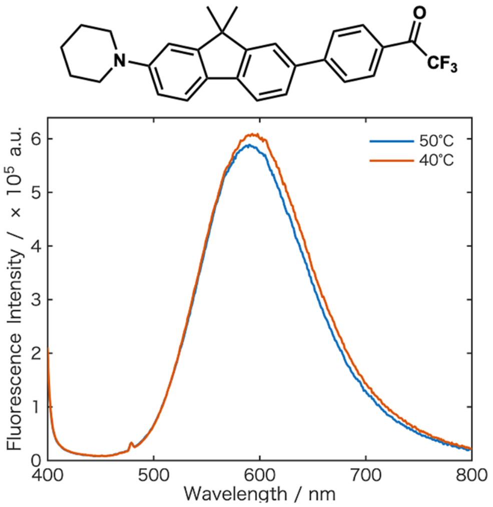

Figure S4. Temperature-dependent fluorescence spectra of **FπF** embedded in a PBMA film, recorded during heating.

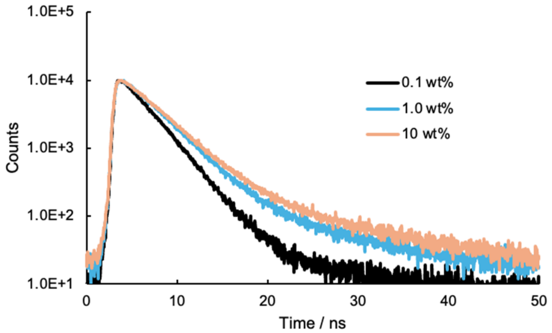

Figure S5. Fluorescence lifetime decay profiles of **FπPCM** embedded in PMMA films at different dye concentrations (0.1, 1.0, and 10 wt%).
